# Population Structure of Clinical and Environmental *Vibrio parahaemolyticus* from the Pacific Northwest Coast of the United States

**DOI:** 10.1371/journal.pone.0055726

**Published:** 2013-02-07

**Authors:** Jeffrey W. Turner, Rohinee N. Paranjpye, Eric D. Landis, Stanley V. Biryukov, Narjol González-Escalona, William B. Nilsson, Mark S. Strom

**Affiliations:** 1 Northwest Fisheries Science Center, National Marine Fisheries Service, National Oceanic and Atmospheric Administration, Seattle, Washington, United States of America; 2 Center for Environmental Genomics, School of Oceanography, University of Washington, Seattle, Washington, United States of America; 3 Center for Veterinary Medicine, Food and Drug Administration, Rockville, Maryland, United States of America; 4 Center for Food Safety and Applied Nutrition, Food and Drug Administration, College Park, Maryland, United States of America; Rockefeller University, United States of America

## Abstract

*Vibrio parahaemolyticus* is a common marine bacterium and a leading cause of seafood-borne bacterial gastroenteritis worldwide. Although this bacterium has been the subject of much research, the population structure of cold-water populations remains largely undescribed. We present a broad phylogenetic analysis of clinical and environmental *V. parahaemolyticus* originating largely from the Pacific Northwest coast of the United States. Repetitive extragenic palindromic PCR (REP-PCR) separated 167 isolates into 39 groups and subsequent multilocus sequence typing (MLST) separated a subset of 77 isolates into 24 sequence types. The Pacific Northwest population exhibited a semi-clonal structure attributed to an environmental clade (ST3, N = 17 isolates) clonally related to the pandemic O3:K6 complex and a clinical clade (ST36, N = 20 isolates) genetically related to a regionally endemic O4:K12 complex. Further, the identification of at least five additional clinical sequence types (i.e., ST43, 50, 65, 135 and 417) demonstrates that *V. parahaemolyticus* gastroenteritis in the Pacific Northwest is polyphyletic in nature. Recombination was evident as a significant source of genetic diversity and in particular, the *rec*A and *dtd*S alleles showed strong support for frequent recombination. Although pandemic-related illnesses were not documented during the study, the environmental occurrence of the pandemic clone may present a significant threat to human health and warrants continued monitoring. It is evident that *V. parahaemolyticus* population structure in the Pacific Northwest is semi-clonal and it would appear that multiple sequence types are contributing to the burden of disease in this region.

## Introduction

*Vibrio parahaemolyticus* is a Gram stain-negative bacterium autochthonous to marine and estuarine environments worldwide [1]–[3]. While the majority of environmental strains are innocuous members of the marine microbiota, small subpopulations are opportunistic pathogens of humans [4]. Potentially virulent strains are commonly differentiated from likely avirulent strains by the presence of the thermostable direct (*tdh*) and *tdh*-related (*trh*) hemolysin genes [5], [6]. Acute gastroenteritis is the most common manifestation of illness and often associated with the consumption of raw or undercooked oysters, which can bioaccumulate the bacterium through filter-feeding [7]–[9].

*V. parahaemolyticus* is a genetically and serotypically diverse species. Outbreaks prior to 1996 were geographically isolated and associated with a diversity of serotypes [10], [11]. Beginning in southeast Asia in 1996, a variant of an existing *V. parahaemolyticus* serotype (O3:K6) was implicated as the cause of larger and less localized outbreaks [12], [13]. Since 1996, numerous outbreak investigations have detailed the emergence, clonal expansion and global dissemination of this O3:K6 serotype [14]–[20]. The O3:K6 serotype and its related serovariants, now recognized as a pandemic clonal complex, have since been associated with a dramatic increase in *V. parahaemolyticus* infections worldwide [9].

In the United States (US), the pandemic serotype (O3:K6) was first reported in 1998 in association with the largest *V. parahaemolyticus* outbreak in US history [21]. Since 1998, an increased incidence of *V. parahaemolyticus* outbreaks in the Pacific Northwest (PNW) region of the United States has coincided chronologically with the introduction of the pandemic strain [22]. However, outbreaks in the PNW have been associated with strains serotypically (O4:K12, O6:K18, O1:K56, O4:K63, O3:K36, O12:K12) and genetically distinct from the O3:K6 serotype [23]–[28]. Elevated *V. parahaemolyticus* case rates in this region have prompted the Washington State Department of Health (WDOH) and the oyster industry to implement strict post-harvest treatment and handling regimens; however, elevated case rates persist in spite of improved post-harvest control measures [22].

Previous studies have utilized multilocus sequence typing (MLST) to successfully examine the genetic diversity of global isolate collections [10],[26],[29] as well as geographically restricted populations [4],[30]–[33]. In the first part of this investigation, we report the highly efficient and discriminatory repetitive extragenic palindromic PCR (REP-PCR) analysis of a large collection of *V. parahaemolyticus* isolates from the PNW. Secondly, we report the reproducible and scalable MLST analysis of a subset of isolates pre-selected by REP-PCR, taking full advantage of the PubMLST database (http://pubmlst.org/vparahaemolyticus/) to investigate clonal and phylogenetic relatedness in a more global context.

The objective of this investigation was to clearly define the phylogenetic relatedness of *V. parahaemolyticus* strains originating from the PNW. We predict the results of this study will inform future efforts to detect pathogenic strains and forecast disease outbreaks. The significance of this work is highlighted by the size and importance of the shellfish industry in Washington State, which according to the Pacific Coast Shellfish Growers Association (http://www.pcsga.net/) produces approximately 75 million pounds of shellfish annually, contributing nearly $110 million to the region's economy.

## Materials and Methods

### Bacterial isolates

One hundred and sixty-seven *V. parahaemolyticus* isolates, obtained from clinical (N = 98) and environmental (N = 69) sources were included in this analysis (see Table S1 for environmental sources and collection dates). The majority of isolates (clinical and environmental) (N = 144) originated from the cold temperate Pacific Northwest (PNW) region of the United States (US) (i.e., Washington State). However, for global perspective we included twenty-three additional isolates, including the O3:K6 pandemic type strain (RIMD2210633) [34] as well as isolates from food-borne outbreaks in the US (Texas, Idaho, Connecticut and New York), Thailand, Vietnam, Bangladesh, Japan and Maldives. Clinical isolates from Washington State were isolated from patients suffering from gastroenteritis traced back to the consumption of raw oysters and obtained through collaborations with the Washington State Department of Health's Public Health Laboratory (WDOH-PHL), the Food and Drug Administration's Pacific Regional Laboratory Northwest (FDA-PRLN) and Gulf Coast Seafood Laboratory (FDA-GCSL), or purchased from the American Type Culture Collection (ATCC) (Manassas, VA, US). Environmental isolates originated from a variety of sources including water, sediment, oysters, clams and plankton net tows. The majority of environmental isolates (N = 59) were recovered from oyster growing areas in Hood Canal and the Washington coast during a *V. parahaemolyticus* monitoring program conducted by our laboratory from June through September 2007 (NOAA's Northwest Fisheries Science Center, NWFSC, Seattle, WA, US). Environmental isolates were isolated at the NWFSC by means of direct plating on thiosulfate-citrate-bile-salts-sucrose agar (TCBS) (BD, Franklin Lakes, NJ, US) and analyzed for the presence of *tlh*, *tdh*, *trh* and urease R (*ure*R) [22] following previously published protocols [5],[35]. Isolates were stored in glycerol (25% final concentration) at −80°C, and grown overnight (16–20 hours) in tryptic soy broth (TSB) (BD, Franklin Lakes, NJ, US) (1.7% casein, 0.3% peptone, 2.0% NaCl, 0.25% phosphate) at 30°C with shaking (150 rpm).

Oyster, water and plankton (net tow) samples were collected by the Washington State Department of Health (WDOH) during routine shellfish monitoring conducted by the Washington State Office of Shellfish and Water Protection. All necessary sampling permits and permissions were obtained by the WDOH. Sample collection did not involve endangered or protected species. The NWFSC Microbiology laboratory is an approved biosafety level 2 facility (BSL-2). Requisite forms and permits pertaining to the acquisition of clinical isolates were completed in accordance with the various laboratories described above (WDOH-PHL, FDA-PRLN, FDA-GCSL and ATCC).

### DNA isolation

DNA was isolated using Qiagen's QIAamp DNA Mini kit in accordance with the manufacturer's instructions (Qiagen Inc., Valencia, CA, US). Isolated DNA was quantified using a Nanodrop spectrophotometer (Nanodrop Products, Wilmington, Delaware, US), diluted to a standardized concentration (∼10 ng/µl) in 1× T low E buffer (10 mM tris, 0.1 mM EDTA, pH 8.0) and stored at −20°C.

### REP-PCR

Repetitive extragenic palindromic PCR (REP-PCR) was performed on the 167 isolates (see Table S1 for isolate identifications) as described previously [36] using a BioRad iCycler (BioRad Inc., Hercules, CA, US) with the primers REP-1D, 5′-NNN RCG YCG NCA TCM GGC-3′, and REP-2D, 5′-RCG YCT TAT CMG GCC TAC-3′, where M is A or C, R is A or G, Y is C or T, and N is any nucleotide. PCR products were resolved by gel electrophoresis (1.5% agarose) buffered in Tris acetate EDTA (TAE) at 80 V for 2 hours, stained with ethidium bromide and visualized under UV using a Fotodyne imaging system (Fotodyne Inc., Hartland, WI, US). REP-PCR fingerprints were analyzed using the BioNumerics software package (Version 6.6, Applied Maths Inc., Sint-Martens-Latem, Belgium). Following conversion, normalization, and background subtraction, the level of similarity between fingerprints was calculated using the Dice coefficient at 1.0% band position tolerance. A representative dendrogram was constructed in BioNumerics using the unweighted pair group method with arithmetic mean (UPGMA). A numerical index of discrimination (D) was determined empirically to compare discriminatory power between REP-PCR and MLST typing methods [37].

### Multilocus sequence typing (MLST)

In accordance with the seven-loci MLST scheme described previously [26], we analyzed *dna*E (DNA polymerase III, α subunit), *gyr*B (DNA gyrase, subunit β), *rec*A (recombinase A), *dtd*S (threonine 3-dehydrogenase), *pnt*A (transhydrogenase, α subunit), *pry*C (dihydro-orotase) and *tna*A (tryptophanase). The selection of 77 isolates from clinical (N = 38) and environmental (N = 39) sources was informed by the REP-PCR analysis of the 167 isolates described in Table S1. Primers specific to these loci were “5′ tailed” with M13 universal primers (forward 5′-TGTAAAACGACGGCCAGT-3′ and reverse 5′-CAGGAAACAGCTATGACC-3′). Primer sequences and conditions for PCR amplification (reagent concentrations and temp-cycling conditions) are available at http://pubmlst.org/vparahaemolyticus/info/protocol.shtml. Amplification was catalyzed with Fusion high-fidelity DNA polymerase (New England Biolabs Inc., Ipswitch, MA, US). PCR products were resolved by gel electrophoresis (1.5% agarose) buffered in Tris acetate EDTA (TAE) at 100 V for 1 hour, stained with ethidium bromide and visualized under UV using a Fotodyne imaging system (as described above). Single amplicon PCR products were purified using Qiagen's Mini Elute PCR Purification Kit in accordance with the manufacturer's instructions (Qiagen Inc., Valencia, CA, US). Purified product was quantified using the Nanodrop spectrophotometer and diluted to ∼10 ng/µl in sterile nuclease-free PCR water. Cycle sequencing was carried out using M13 universal primers and Applied Biosystem's (ABI) BigDye Terminator (BDT) v3.1 Cycle Sequencing Kit (Life Technologies Corp., Carlsbad, CA, US). Reactions (5 µl) contained 0.5 µl of 5× BDT buffer (0.5× final concentration), 0.4 µl of 10 µM M13 primer (forward or reverse) (0.8 µM final concentration), 1 µl BDT Ready Reaction Mix, 2 µl of purified DNA template and 1.1 µl nuclease-free PCR water. Cycling was carried out on a BioRad iCycler (BioRad Inc., Hercules, CA, US) with an initial denaturation of 96°C for 1 min, followed by 25 cycles of denaturation at 96°C for 10 s, primer annealing at 50°C for 5 s, and extension at 72°C for 4 min. Excess dye-terminator was removed using Agencourt's CleanSEQ system in accordance with the manufacturer's instructions (Beckman Coulter Inc., Danvars, MA, US). Sequencing was performed on ABI's PRISM 3100 Genetic Analyzer. DNA trace sequences were inspected and assembled using MacVector version 12.0.4 (MacVector Inc., Cary, NC, US).

### Loci statistics

To evaluate the potential that the loci used in our typing schemes were subject to varying degrees of selection, we calculated the number of alleles, number of polymorphic sites and nucleotide diversity per site (π) using DnaSP version 5 [38] The ratio of nonsynonymous to synonymous substitutions (*d*_N_/*d*_S_) was calculated by the Nei and Gojobori method [39] as implemented in START version 2 [40]. This statistic is a measure of selection where a *d*_N_/*d*_S_<1 indicates purifying selection, a *d*_N_/*d*_S_ = 1 indicates neutral selection and a *d*_N_/*d*_S_>1 indicates positive selection.

### Assignment to sequence types

Alleles were queried against the PubMLST *V. parahaemolyticus* database (http://pubmlst.org/vparahaemolyticus/) to determine the allelic profile and sequence type (ST) for each isolate. A numerical index of discrimination (D) was determined empirically to compare discriminatory power between REP-PCR and MLST typing methods [37].

### Phylogenetic relatedness

Multiple sequence alignments (MSAs) for each locus were aligned in MUSCLE [41] and trimmed using trimAL [42]. Statistical models of nucleotide substitution were determined in jModelTest [43] using the Akaike Information Criterion (AIC). A majority consensus phylogeny of the concatenated loci (3,682 bp) was constructed using the Bayesian Markov chain Monte Carlo (MCMC) method as implemented in MrBayes version 3.2 [44]. Concatenated loci were partitioned such that pre-determined models of nucleotide substitution (jModelTest above) were applied to each locus and evolutionary rates were allowed to vary between loci using a flat Dirichlet prior distribution. Two independent MCMC runs were repeated for 1,000,000 generations and sampled every 5,000 generations. Convergence was assured via the standard deviation of split frequencies (<0.05) and the potential scale reduction factor (PSRF∼1). The resulting MrBayes cladogram and associated posterior probabilities for each split were illustrated using FigTree version 1.3.1 and edited in Pixelmator version 2.1.

### Assignment to clonal complexes

Assignment of sequence types (STs) to clonal complexes was accomplished using eBURST version 3 (http://eburst/mlst.net) as described previously, using 1,000 bootstrap resamplings (15). Inclusion in a clonal complex was restricted to STs sharing all 7 alleles as well as single locus variants (SLV), which share at least 6 of the 7 alleles. Double locus variants (DLV), defined as STs sharing 5 of 7 alleles, were not assigned as members of a clonal complex.

### Estimates of recombination

The contribution of recombination and mutation to clonal diversity was calculated empirically (by visual inspection) as the per-allele and per-site recombination/mutation (r/m) parameter as described previously [45] Briefly, any SLV arising from a single nucleotide polymorphism (SNP) (not reproduced in the population) was considered to have arisen by mutation while any SLV arising from multiple SNPs (reproduced in the population) was considered to have arisen by recombination. Given a non-redundant list of all allelic profiles, LIAN version 3.5 [46] was used to calculate the standard index of association (I^A^_S_). This statistic describes the linkage disequilibrium in a multilocus data set where a high rate of recombination (relative to mutation rates) is indicative of equilibrium (I_A_∼0) and a low rate of recombination is indicative of linkage disequilibrium (I_A_>1) [46]. We utilized a NeighborNet network as implemented in Splitstree version 4 [47] to evaluate the impact of recombination and validate the results of phylogenetic relatedness (above). Evidence of recombination was further evaluated by calculating the Pairwise Homoplasy Index (φ_w_) [48] for the NeighborNet network (above) as implemented in Splitstree. To detect evidence of intragenic recombination, individual loci were also analyzed by the Pairwise Homoplasy Index (φ_w_) [48] in addition to the Sawyer's Run Test [49] as implemented in START [40].

### Nucleotide sequence accession numbers

Gene sequences for *dnaE*, *gyrB*, *recA*, *dtdS*, *pntA*, *pryC* and *tnaA* were deposited in GenBank (accession numbers JQ958991 to JQ959536). Isolate descriptions (such as origin and date of isolation), allelic profiles and sequencing traces (unique alleles) are available in the PubMLST database (http://pubmlst.org.vparahaemolyticus/).

## Results

### REP-PCR

**Figure 1:**
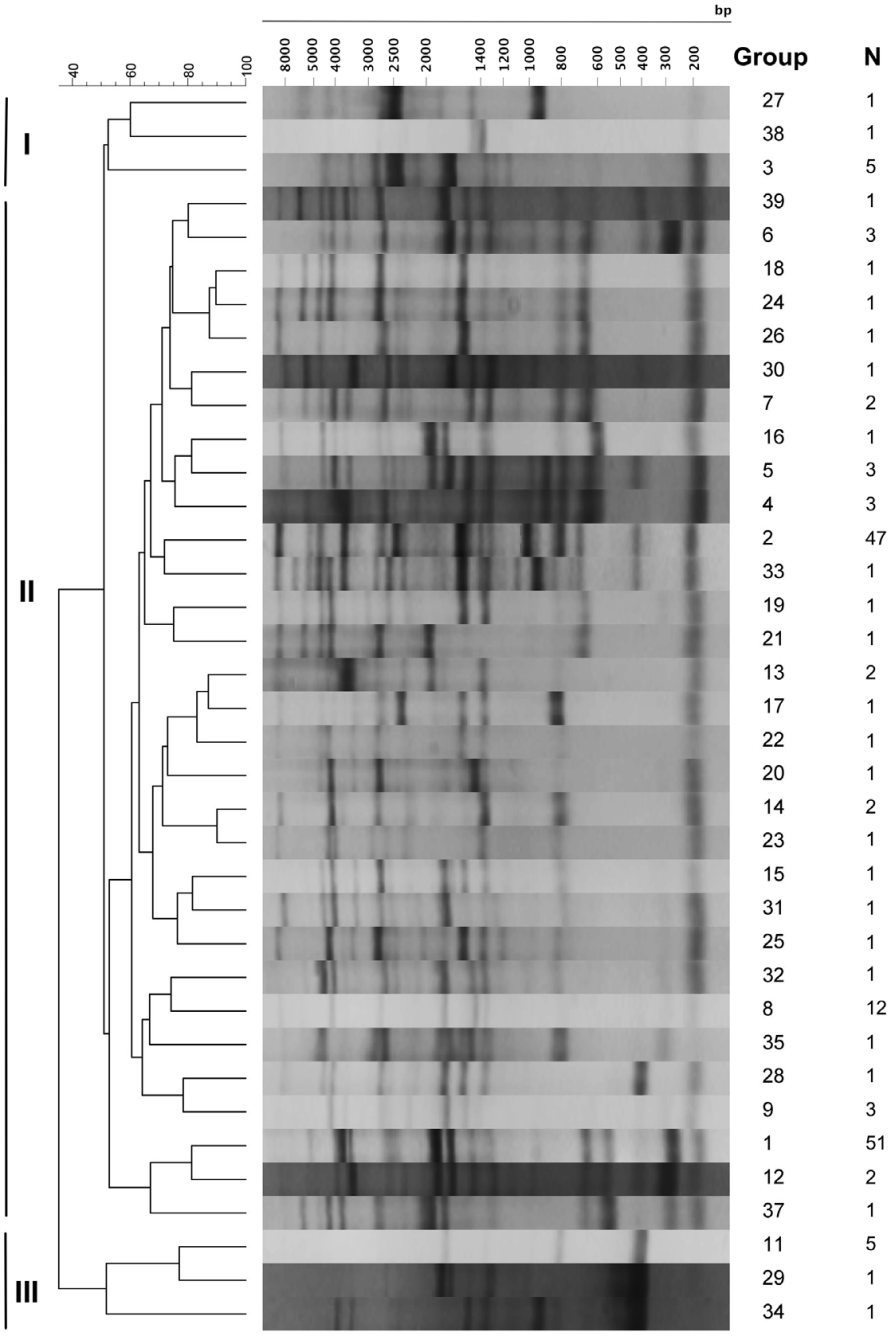
REP-PCR patterns and representative dendrogram. The electrophoresis banding patterns of 167 *V. parahaemolyticus* isolates assayed by REP-PCR is shown. BioNumerics analysis of patterns revealed 39 unique REP-PCR groups comprised of N isolates. The corresponding BioNumerics dendrogram illustrates the genetic relatedness between REP-PCR groups, which we grouped into three major clusters (I, II, III). Groups 27, 28 and 3 comprise cluster I while groups 11, 29 and 34 comprise cluster III and all remaining groups comprise cluster II. Electrophoresis banding patterns shown with scale indicating fragment size in base pairs (bp).

REP-PCR proved an effective method of screening a large number of environmental and clinical isolates and informed the selection of isolates for MLST analysis. The 167 isolates analyzed by REP-PCR separated into 39 groups with the majority of isolates divided among three major clusters: cluster I (groups 27, 38 and 3), cluster III (groups 11, 29 and 34) and cluster II (containing all additional groups) (Figure 1). In cluster II, group 1 (51 isolates) and group 2 (47 isolates) accounted for the majority of isolates (98/166). Further, cluster II exhibited the highest level of diversity, separating into 33 groups. Group 1 was primarily composed of clinical isolates from Washington State (N = 48), while the remaining isolates (N = 3) originated from oysters harvested in shellfish growing areas in Washington State (see Table S2). All group 1 isolates were PCR positive for the thermostable direct hemolysin (*tdh*), the thermostable related hemolysin (*trh*) and urease R (*ure*R). Group 2 was composed of 34 environmental isolates and 13 clinical isolates, including the prototypical pandemic strain (RIMD2210633) and 12 additional clinical pandemic isolates. All group 2 isolates were *tdh* positive, but variable for *trh* and *ure*R. Groups 3–7 included between 2 to 5 environmental isolates each and were variable for the presence of *tdh*, *trh* and *ure*R. Group 8 included 12 isolates (11 clinical and 1 environmental), all of which carried *tdh*, *trh* and *ure*R. Groups 9–14 included mostly clinical isolates (except isolate AOC1 in group 10), which were again variable for the presence of *tdh*, *trh* and *ure*R. Isolates in groups 15 to 39 included single isolates with unique profiles and were also variable for the presence of *tdh*, *trh* and *ure*R (see Table S2). The discrimination index for the REP-based phylogeny was 0.821.

### Loci statistics

A subgroup of the 167 REP-PCR isolates (N = 77) was selected to represent a diversity of sources, dates of isolation and REP groups, and analyzed by MLST. Table 1 lists the chromosome location, loci, internal fragment size, number of alleles, number of polymorphic sites, rate of nonsynonymous (*d*_N_) and synonymous (*d*_S_) substitutions, and *d*_N_/*d*_S_ for each locus (*dna*E, *gyr*B, *rec*A, *dtd*S, *pnt*A, *pyr*C and *tna*A). The 7 loci were divided between chromosome I (*dna*E, *gyr*B, *rec*A) and II (*dtd*S, *pnt*A, *pyr*C, *tna*A) based on the complete genome sequence of *V. parahaemolyticus* RIMD2210633 (22). Among all loci, internal fragment size ranged from 423 bp (*tna*A) to 729 bp (*rec*A). The number of alleles ranged from 14 (*pnt*A) to 21 (*rec*A). The number of polymorphic sites ranged from 22 (*pnt*A) to 51 (*rec*A). The nucleotide diversity (π) ranged from 0.0103 (*gyr*B) to 0.0206 (*rec*A). Rates of nonsynonymous mutations (*d*_N_) ranged from 0.0000 (*dtd*S) to 0.0022 (*pyr*C) and rates of synonymous mutations (*d*_S_) ranged from 0.0462 (*pyr*C) to 0.1160 (*rec*A). The ratio of nonsynonymous to synonymous mutations showed that all loci were subject to purifying selection (i.e., *d*_N_/*d*_S_ ratios were <1 for each locus, Table 1).

**Table 1:** Summary of loci statistics included in the MLST scheme (*dna*E, *gyr*B, *rec*A, *dtd*S, *pnt*A, *pry*C and *tna*A) such as fragment length, number of alleles and polymorphic sites, nucleotide diversity (π), rates of nonsynonymous (*d*_N_) and synonymous (*d*_S_) mutations, and tests of selection (*d*_N_/*d*_S_).

Chromosome	Locus	Fragment length (bp)	Alleles	Polymorphic sites	π	*d*_N_ a	*d*_S_ a	*d*_N_/*d*_S_ a
I	*dna*E	557	19	30	0.0120	0.0013	0.0584	0.0223
	*gyr*B	592	18	32	0.0103	0.0002	0.0559	0.0044
	*rec*A	729	21	51	0.0206	0.0007	0.1160	0.0059
II	*dtd*S	458	18	34	0.0192	0.0000	0.0979	0.0000
	*pnt*A	430	14	22	0.0107	0.0017	0.0557	0.0313
	*pyr*C	493	19	26	0.0112	0.0022	0.0462	0.0482
	*tna*A	423	17	23	0.0118	0.0015	0.0522	0.0278

a Rate of nonsynonymous (*d*_N_) and synonymous (*d*_S_) substitutions where *d*_N_/*d*_S_<1 indicates that the loci is subject to purifying selection.

### Assignment of sequence types

A query of allelic profiles against the PubMLST database (http://pubmlst.org/vparahaemolyticus/) revealed a total of 24 sequence types (STs), including the presence of 3 new STs (ST416, isolate 204; ST417, isolates 3631 and 3646; ST418, isolate 2006286, see Table S3). New STs were based on the identification of 1 new allele (*pyr*C) in isolate 204 and 4 new alleles (*rec*A, *dtd*S, *pnt*A and *pyr*C) in isolate 3631 and 3646 (see Table S3). The exceptional ST418 was based on a new combination of alleles, all of which have been identified previously. The majority of isolates were divided among 9 sequence types: ST3 (N = 20), ST36 (N = 20), ST43 (N = 6), ST 34 (N = 4), ST65 (N = 3), ST135 (N = 2), ST137 (N = 3), ST138 (N = 2) and ST416 (N = 2). The remaining 15 STs were represented by single isolates. In agreement with REP-PCR groups, ST3 included the 4 pandemic O3:K6 representative isolates (RIMD2210633, TX2103, BE98-2029 and AP-14861). The remaining ST3 isolates (N = 16) originated from environmental sources. Conversely, ST36 (N = 20), ST43 (N = 6), ST65 (N = 3) and ST417 (N = 2) represented the majority of clinical isolates from Washington State. The discrimination index (D) for the MLST-based phylogeny was 0.859.

### Phylogenetic relatedness

**Figure 2:**
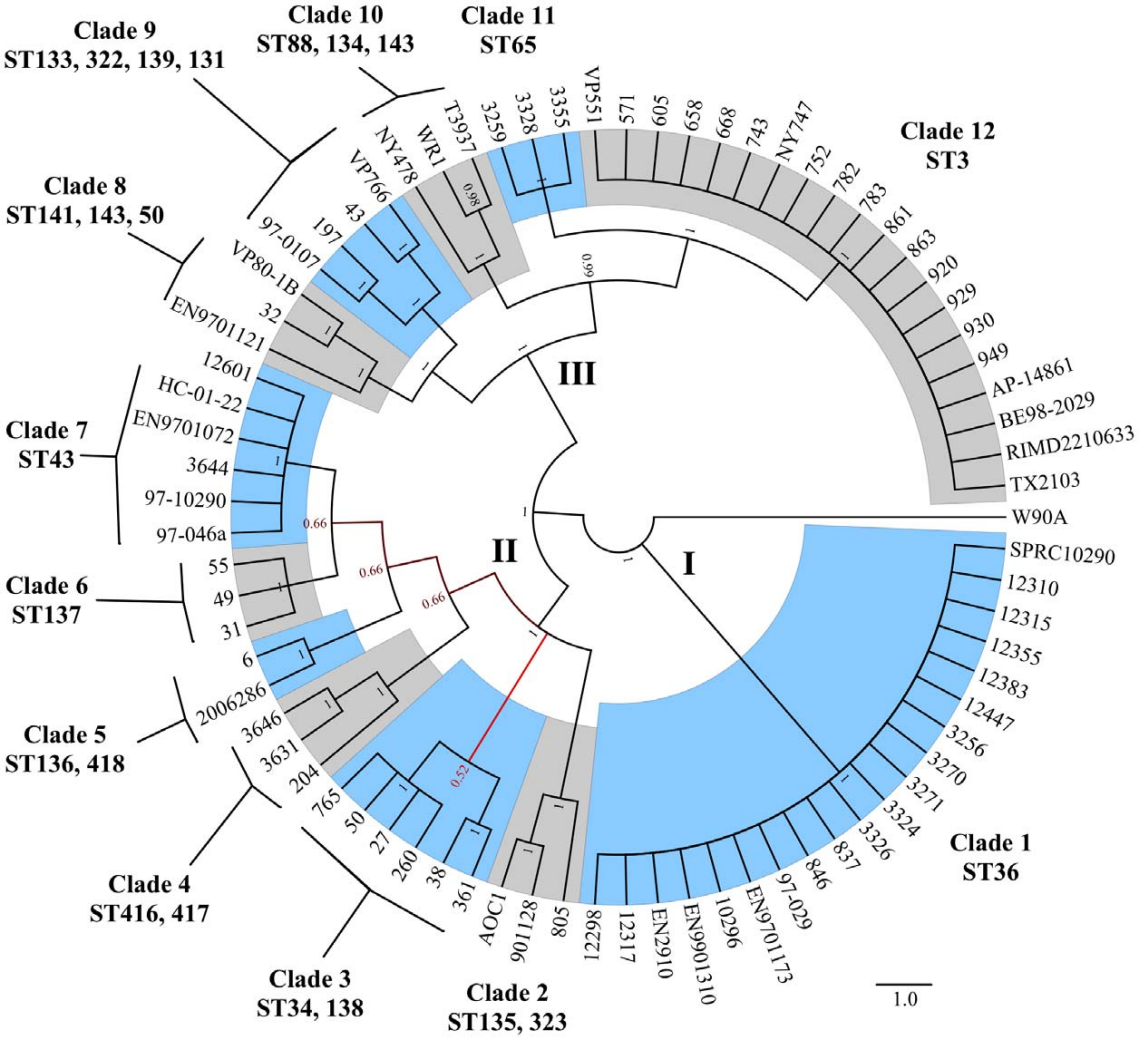
MLST majority consensus phylogeny. A majority consensus phylogeny of 77 *V. parahaemolyticus* isolates based on 7 concatenated housekeeping loci (*dna*E, *gyr*B, *rec*A, *dtd*S, *pnt*A, *pry*C and *tna*A) and representing 3,682 total nucleotides was constructed using the Bayesian Markov chain Monte Carlo (MCMC) method as implemented in MrBayes v3.2. The 77 isolates included in this phylogeny were separated into three major clusters (I, II, III) and 12 distinct clades (1–12). Sequence typing (ST) designations for MLST analysis describe the 24 MLST sequence types comprising each of the 12 clades. Distinct clades clearly highlighted by alternating blue and gray shading. Nodes are labeled with posterior probabilities (0–1) while cladogram shading is indicative of branches with weak support (red) and strong support (black).

A majority consensus phylogeny was constructed from the concatenated loci (Figure 2). Loci were partitioned to permit the application of different DNA substitution models such that K80+G [50] was applied to *dna*E, *gyr*B, *rec*A, *tna*A, K80+I+G [50] was applied to *dtd*S, F81+G [51] was applied to *pnt*A, and JC+G [52] was applied to *pyr*C. Posterior probabilities (0 to 1) were illustrated as a color gradient between red (weak support) and black (strong support). Clades were distinguished by alternating blue and gray highlighting. The 77 isolates included in this phylogeny were separated into three major clusters (I, II, III) and 12 distinct clades (1–12) (Figure 2). Cluster I (N = 20 isolates) was the most homogenous and composed of only one clade (clade 1) and only one sequence type (ST36) (Figure 2). Clade 1 comprised 18 clinical isolates and 2 environmental isolates (see Table S3). All clade 1 isolates were PCR positive for the thermostable direct hemolysin (*tdh*), the thermostable related hemolysin (*trh*) and urease R (*ure*R). Cluster II (N = 23 isolates) was composed of six clades (2–7) and ten sequence types (ST34, 43, 135, 136, 137, 138, 323, 416, 417 and 418) (Figure 2). Cluster II comprised 9 clinical isolates and 14 environmental isolates (see Table S3). Within cluster II, clade 3 was composed of environmental isolates (N = 6) while clade 7 (N = 6) was primarily composed of clinical isolates (N = 5). Clades 2 (N = 3), 4 (N = 3) and 5 (N = 2) comprised clinical and environmental isolates while clade 6 (N = 3) comprised only environmental isolates. Cluster III (N = 33 isolates) was composed of five clades (8–12) and 12 sequence types (ST3, 50, 65, 88, 131, 133, 134, 139, 141, 143 and 322) (Figure 2). Cluster III comprised 23 clinical isolates and 10 environmental isolates. Within cluster III, clades 8 (N = 3), 10 (N = 3) and 12 (N = 20) were composed of clinical and environmental isolates (see Table S3). Interestingly, clade 12 comprised sixteen environmental isolates from various sources (water, oysters and plankton net tows) and four clinical pandemic O3:K6 isolates (RIMD2210633, TX2103, BE982029 and AP14861) isolated from geographically distant outbreaks of *V. parahaemolyticus* gastroenteritis. Clade 9 (N = 4) was composed of environmental isolates while clade 11 (N = 3) was composed of clinical isolates.

### Assignment to clonal complexes

A global eBURST analysis of all STs (the 24 presented in this study and the 415 collected in the *V. parahaemolyticus* PubMLST database (http://pubmlst.org/vparahaemolyticus/) revealed the 24 STs belong to 6 clonal complexes (CCs), 4 groups and 14 singletons (see Table S3). The STs belonging to a clonal complex included ST3 (CC3), ST34 (CC34), ST50 (CC50), ST88 (a CC with multiple candidate founders), ST133 and ST322 (CC322) and ST418 (CC110). Among these clonal complexes, this analysis revealed only two single locus variants (SLVs) (ST133 and 322). The alleles giving rise to these SLVs were the *gyr*B alleles of isolates 43 (ST322) and VP766 (ST133) which differed at 8 nucleotide sites. All 8 nucleotide variations between these 2 alleles were present in other *gyr*B alleles, suggesting that these SLV arose from recombination rather than point mutation. Additionally, we detected two pairs of double locus variants (DLVs) (ST36 and ST59; ST141 and ST142). Clonal complex 3 (CC3) remains the most populated clonal complex in the MLST database (N = 174 isolates); however, the addition of this data expanded the number of isolates in ST36 from 7 to 27.

### Estimates of recombination

**Figure 3:**
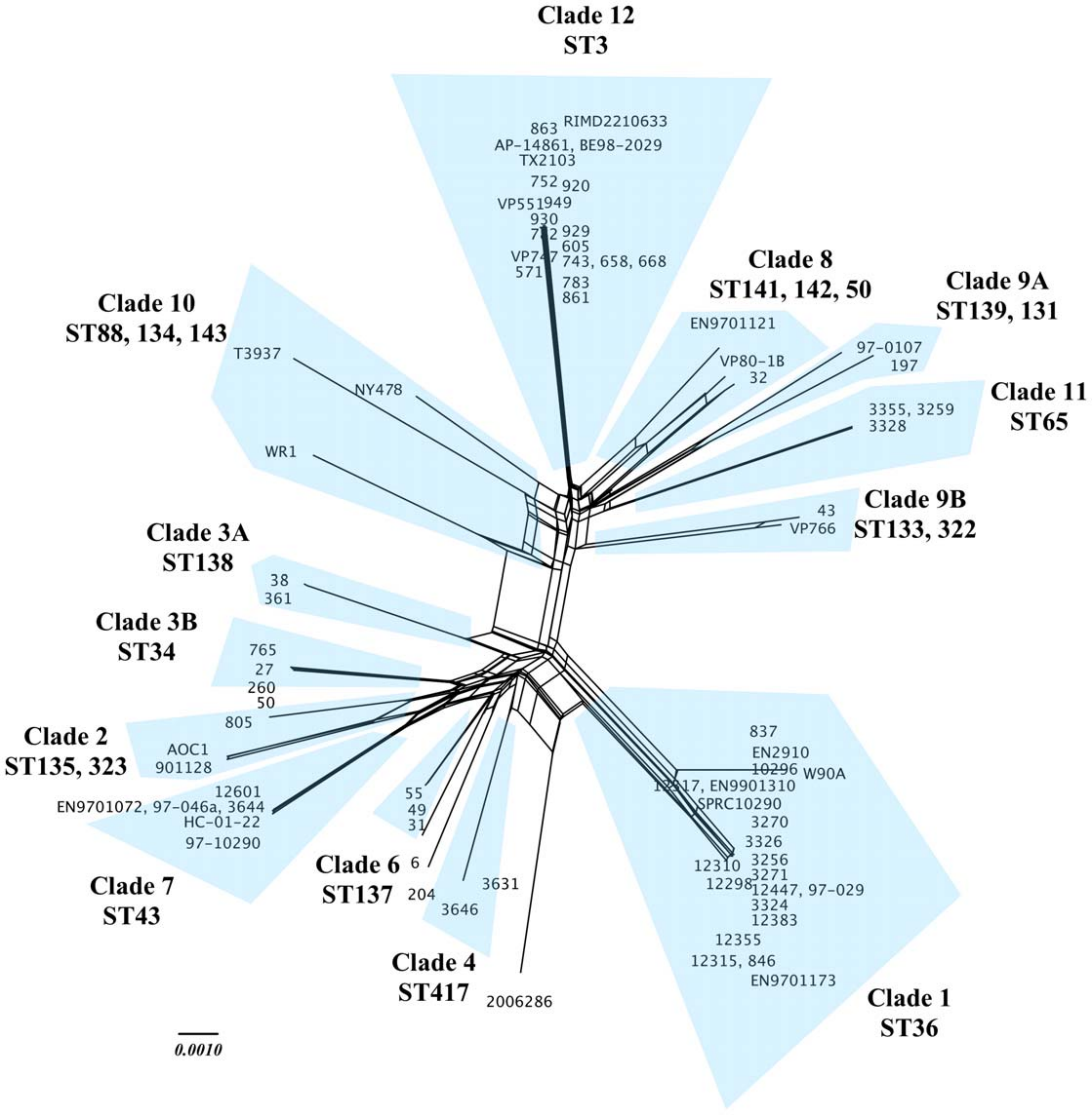
NeighborNet analysis. SplitsTree v4 NeigborNet analysis of 77 *V. parahaemolyticus* isolates based on 7 concatenated housekeeping loci (*dna*E, *gyr*B, *rec*A, *dtd*S, *pnt*A, *pry*C and *tna*A) representing a total 3,682 nucleotides. Sequence typing (ST) designations for MLST analysis and phylogenetic clades (1–12) included for reference. Regions of the network showing extensive reticulation (e.g., clades 8 and 10), consistent with higher rates of recombination, contrast with the less reticulated nature of clade 12. Highlights in blue distinguish groups of isolates sharing ST and clade designations and function to facilitate comparison with Figure 2.

The relative contribution of recombination and mutation (r/m) to clonal diversification among SLVs resulted in a per-allele r/m parameter of 2∶0 and a per-site r/m parameter of 8∶0. LIAN tests for recombination returned a “standardized” index of association (I^A^_S_) of 0.1295 (P<0.000), indicating significant linkage disequilibrium and suggesting that allelic variation is non-random. The Pairwise Homoplasy Index (φ_w_), applied to the NeighborNet network based on the concatenated gene set, also confirmed these loci were subjected to a significant rate of recombination (mean = 0.3665, P<0.001). Similarly, φ_w_ and Sawyer's tests showed strong support for recombination among *rec*A and *dtd*S alleles (P<0.05), and weak support among *dna*E, *pyr*C and *tna*A alleles (P<0.05) (Table 2). The Splitstree NeighborNet network revealed strong support for some clades (i.e., clades 1 and 12) as evidenced by a lack of reticulated structure associated with those clades (Figure 3). However, a more reticulate structure is evident for additional clades (e.g., clades 3, 4, 6 and 7), suggesting that recombination plays a stronger role among those clades. Discrepancies between the NeighborNet network and Bayesian phylogeny were evident by splits dividing clade 3 (3A and 3B) and clade 9 (9A and 9B). Similarly, a split separated isolates 2006286 and 204 leaving clade 5 unresolved (Figure 3).

**Table 2:** Results of the Pairwise Homoplasy Index (φ_w_)^a^ and Sawyer’s Run Test^b^ for homologous recombination performed on individual loci included in the MLST scheme (*dna*E, *gyr*B, *rec*A, *dtd*S, *pnt*A, *pry*C and *tna*A).

Chromosome	Locus	φ_w_ a (P)*	SSCF^b^ (P)*	SSUF^b^ (P)*
I	*dna*E	0.1231 (0.061)	28,193 (0.040)	17,852,729 (0.008)
	*gyr*B	0.1779 (0.163)	25,202 (0.085)	13,820,083 (0.080)
	*rec*A	0.2526 (0.000)	92,754 (0.000)	24,289,938 (0.000)
II	*dtd*S	0.4359 (0.000)	29,905 (0.000)	6,896,551 (0.016)
	*pnt*A	0.0784 (0.293)	6,455 (0.319)	4,917,051 (0.671)
	*pyr*C	0.1666 (0.012)	17,084 (0.063)	15,456,797 (0.055)
	*tna*A	0.0994 (0.024)	11,572 (0.140)	7,846,412 (0.128)

a mean Pairwise Homoplasy Index, see reference [48].

b sum of squared lengths of condensed fragments (SSCF) and sum of squared lengths of uncondensed fragments (SSUF), see reference [49].

* significance declared at P<0.05.

## Discussion

We present the REP-PCR and MLST-based analysis of *V. parahaemolyticus* population structure among clinical and environmental isolates originating from the cold temperate PNW region of the US. In general, clinical and environmental isolates exhibited a semi-clonal structure with 167 isolates separating into 39 REP-PCR groups, while subsequent MLST analysis on a subset of 77 isolates identified 24 sequence types. The identification of multiple clinical STs (e.g., 36, 43, 50, 65, 135 and 417) demonstrates that *V. parahaemolyticus* gastroenteritis in the PNW is polyphyletic in nature. Additionally, the discovery of an environmental complex (ST3) clonally related to the pandemic complex may pose a significant public health threat and further confirms that the environment is a reservoir of virulent strains.

REP-PCR proved to be an effective and discriminatory tool for screening a large number of isolates. REP patterns informed the selection of a subset of isolates for a targeted MLST investigation, which allowed comparison to a global database and an estimation of phylogenetic diversity. Discrimination indexes of the REP-PCR and targeted MLST analyses were 0.821 and 0.859 respectively. REP-PCR and MLST showed strong agreement and only minor discrepancies in that each technique discriminated the subset of 77 isolates into 24 REP-PCR groups and 24 multilocus sequence types, respectively. Discrepancies between the two techniques were limited to isolates W90A (REP group 1 and ST59) and EN9901310 (REP group 12 and ST36). Based on our phylogenetic analyses, W90A is a unique isolate while EN9901310 groups with the closely related ST36 (clade 1).

Previous MLST-based studies have demonstrated that *V. parahaemolyticus* populations can be extremely diverse [4],[26],[29] even within a single geographic locality [4] [30],–[33]. The eBURST algorithm revealed that the 24 STs described in this study belong to 6 clonal complexes, 4 groups and 14 singletons. The discovery of only two SLVs suggests a general absence of linkage between STs and supports the hypothesis that these complexes, groups and singletons are genetically exclusive groups. However, phylogenetic analyses revealed that clades 2, 3, 4, 5, 8, 9 and 10 are comprised of related sequence types.

Phylogenetic analysis also illustrated the semi-clonal structure of both clinical and environmental populations. In particular, clinical isolates comprising clade 1 (ST36) and environmental isolates comprising clade 12 (ST3) were highly homogenous. A high degree of homogeneity (i.e., clonality) among clinical isolates is well supported in the literature [4],[10],[26],[29]; however, environmental isolates commonly comprise a more heterogeneous (i.e., non-clonal) population [4],[23],[30]. Although our selection of only potentially virulent (*trh* ^+^ or *tdh* ^+^ or both) isolates may have introduced an artificially high degree of homogeneity, previous studies have shown that even potentially virulent environmental isolates demonstrate heterogeneity. For example, reports of 41 [4] and 91 [23] potentially virulent environmental isolates were characterized as genetically and serotypically heterogeneous. However, in this study, we interpret homogeneity among environmental isolates as a unique observation explained largely by the presence of a single clonal environmental group (clade 12) sharing the same sequence type (ST3) and genotype (*trh* ^−^ and *tdh* ^+^) as the pandemic O3:K6 complex.

According to the PubMLST database, the ST3 clonal complex (N = 172) is largely clinical (149/172) and representative of the pandemic clonal complex. The close phylogenetic relationship between clade 12 and the pandemic complex supports the possible virulence of this environmental clade. Thus, we suggest the absence of pandemic-related illness in the PNW during this study was not due to the absence of the pandemic clonal complex. In fact, isolates clonally related to the pandemic complex (ST3) represented 34.7% (17/49) of the environmental isolates included in this MLST investigation. A recent report of one illness associated with a pandemic isolate (O3:K6) in the summer of 2011 (personal communication with WDOH and FDA) suggests a potential shift in the epidemiology of ST3 isolates in the PNW that warrants continued monitoring. Conversely, the absence of pandemic-related illness may indicate the ST3-related strains described in this study are attenuated or avirulent.

A diversity of sequence types (ST36, 43, 50, 65, 135 and 417) was responsible for *V. parahaemolyticus* illnesses in Washington State. Query against the PubMLST database revealed that 27 isolates share the ST36 allelic profile and the majority of those isolates (N = 20) were deposited as part of this investigation. While ST36 was not determined to be a clonal complex by eBURST analysis due to an absence of SLVs, this ST was highly homogenous and largely clinical (21/27). Taken together, REP-PCR and MLST analyses support the conclusion that ST36 represents a genetically exclusive sequence type. This ST included the clinical reference isolate SPRC10290 (O4:K12) which has served as a reference in several prior investigations [23],[53]–[55]. Based on the limited number of isolates in the PubMLST database, the distribution of ST36 appears to be restricted to the Pacific coast of the US with the majority of those isolates originating from Washington State (19/27). The distribution of ST36 and the inclusion of SPRC10290 suggest this ST is clonally related to the O4:K12 complex cited previously as potentially endemic to the Pacific coast of US and Mexico [28].

According to the limited data provided by the MLST database, the distribution of ST43, which was composed of clinical (N = 5) and environmental isolates (N = 4), also appears to be restricted to the Pacific coast of US. Additional clinical sequence types included two STs first associated with illness in 2005 and 2007, ST65 and ST417. According to the PubMLST database, ST65 is presently composed of six clinical isolates, three of which were deposited as part of this investigation (3355, 3328 and 3259) and associated with illness during an outbreak in Washington State in 2007 (this study). The three additional ST65 isolates were first associated with *V. parahaemolyticus* gastroenteritis in Peru in 2005 and Chile in 2007. A new sequence type (ST417, N = 2) identified in this study was first associated with illness during an outbreak in Washington State in 2006 (this study). Although some sequence types described in this study (ST36, 43, 65, 417) appear to be geographically restricted to the Pacific coast of the Americas (Peru, Chile and or US), these sequence types are underrepresented in the MLST database and may be more widely distributed.

Clinical and environmental isolates comprising ST36 and ST43 were isolated over 10 years (1997–2007), suggesting that local environmental conditions favor the survival and persistence of these STs. Similarly, Abbott et al. [28] concluded that the persistence of the O4:K12 serotype was favored by local ecological factors. Meanwhile, the identification of a new clinical ST (i.e., 417) speaks to the diversity of the species and serves as a reminder that pathogenic strains can emerge from the environmental reservoir. Although environmental isolates originated from a variety of habitats (water, oyster, plankton and sediment), no correlation was observed between habitat and population structure. Similarly, a previous MLST study also reported no correlation between environmental source and phylogenetic relatedness [30], supporting the conclusion that *V. parahaemolyticus* is a generalist in that isolates belonging to the same ST and phylogenetic clade appear to survive and persist in association with a variety of environmental habitats.

Previous MLST investigations have shown that recombination plays a significant role in the introduction of clonal diversity in *V. parahaemolyticus* populations [26],[29],[30],[32]. Visual inspection and calculation of the r/m parameter indicated that SLVs resulted from recombination events. Similarly, significant linkage disequilibrium and significant pairwise homoplasy (φ_w_) was detected among the complete set of allelic profiles and concatenated loci, respectively. Further, φ_w_ and Sawyer's analyses showed strong support for homologous recombination in *rec*A and *dtd*S alleles, weaker support for *dna*E, *pyr*C and *tna*A, and no support for *gyr*B and *pnt*A. In agreement with these results, Yan et al. [29] reported significant support for recombination in *dtd*S and *tna*A while Yu et al. [32] reported high rates of recombination in *rec*A. Increased recombination rates among environmental strains may result from inactivation of mismatch repair (MMR), which has been shown to increase rates of mutation and recombination in *V. parahaemolyticus* [56].

In summary, the *V. parahaemolyticus* population in the PNW appears to be semi-clonal in nature. Further, clonality appears to largely result from the presence of two major homogenous clades. The first is clinical (clade 1, ST36) and related to an endemic complex (O4:K12) while the second is environmental (clade 12, ST3) and related to the clonal pandemic complex (O3:K6). Outside of these homogenous clades, the presence of at least 5 additional clinical STs further complicates the epidemiology of *V. parahaemolyticus* in this region. Current efforts are focused on the sequencing and genomic comparison of 23 *V. parahaemolyticus* isolates included in this study. Central to this genomic endeavor is the characterization of environmental isolates, which share the same sequence type (ST3) and genotype (*trh* ^−^ and *tdh* ^+^) as the pandemic O3:K6 complex.

## Supporting Information

Table S1 ***Vibrio parahaemolyticus*** **isolate description.** Description of the *V. parahaemolyticus* isolates (N = 167) included in this investigation including source (clinical and environmental), location and date of isolation, and laboratory source.
(DOC)

Table S2 **REP-PCR results.** Summary of REP-PCR results (N = 167 isolates) organized by REP group including the number of isolates in each group and the genotype (*tdh*, *trh*, ureR) of each REP-PCR group.
(DOC)

Table S3 **MLST and eBURST results.** MLST results (N = 77 isolates) organized by sequence type (ST) including the determination of whether a given ST is part of a clonal complex (CC), group (G) or singleton (S) as determined by eBURST.
(DOC)
