# Supplementary material for: Population Structure of Clinical and Environmental Vibrio parahaemolyticus from the Pacific Northwest Coast of the United States
Source: PLoS One. 2013 Feb 7;8(2):e55726. doi: 10.1371/journal.pone.0055726 (PMC3567088; doi:10.1371/journal.pone.0055726)
Supplement: Table S1 — Vibrio parahaemolyticus isolate description. Description of the V. parahaemolyticus isolates (N = 167) included in this investigation including source (clinical and environmental), location and date of isolation, and laboratory source. (DOC) [file pone.0055726.s001.doc]

Table S1. *Vibrio parahaemolyticus* isolate description.

| **Isolates** | **Source** | **Origin** | **Laboratory^a^** |
| --- | --- | --- | --- |
| 2 | oyster | WA, 2007 | NWFSC |
| 6 | oyster | WA, 2007 | NWFSC |
| 7 | net tow | WA, 2007 | NWFSC |
| 27 | water | WA, 2007 | NWFSC |
| 31 | oyster | WA, 2007 | NWFSC |
| 32 | oyster | WA, 2007 | NWFSC |
| 38 | oyster | WA, 2007 | NWFSC |
| 43 | oyster | WA, 2007 | NWFSC |
| 49 | oyster | WA, 2007 | NWFSC |
| 50 | water | WA, 2007 | NWFSC |
| 55 | net tow | WA, 2007 | NWFSC |
| 67 | net tow | WA, 2007 | NWFSC |
| 197 | oyster | WA, 2007 | NWFSC |
| 204 | oyster | WA, 2007 | NWFSC |
| 260 | water | WA, 2007 | NWFSC |
| 361 | oyster | WA, 2007 | NWFSC |
| VP551 | water | WA, 2007 | NWFSC |
| 571 | water | WA, 2007 | NWFSC |
| 584 | water | WA, 2007 | NWFSC |
| 585 | water | WA, 2007 | NWFSC |
| 586 | water | WA, 2007 | NWFSC |
| 587 | water | WA, 2007 | NWFSC |
| 588 | water | WA, 2007 | NWFSC |
| 604 | net tow | WA, 2007 | NWFSC |
| 605 | net tow | WA, 2007 | NWFSC |
| 606 | Plankton | WA, 2007 | NWFSC |
| 658 | net tow | WA, 2007 | NWFSC |
| 661 | water | WA, 2007 | NWFSC |
| 668 | water | WA, 2007 | NWFSC |
| 671 | water | WA, 2007 | NWFSC |
| 688 | net tow | WA, 2007 | NWFSC |
| 743 | water | WA, 2007 | NWFSC |
| VP747 | net tow | WA, 2007 | NWFSC |
| 752 | water | WA, 2007 | NWFSC |
| 765 | net tow | WA, 2007 | NWFSC |
| VP766 | net tow | WA, 2007 | NWFSC |
| 782 | net tow | WA, 2007 | NWFSC |
| 783 | net tow | WA, 2007 | NWFSC |
| 805 | water | WA, 2007 | NWFSC |
| 837 | oyster | WA, 2007 | NWFSC |
| 846 | oyster | WA, 2007 | NWFSC |
| 861 | water | WA, 2007 | NWFSC |
| 863 | net tow | WA, 2007 | NWFSC |
| 864 | net tow | WA, 2007 | NWFSC |
| 865 | net tow | WA, 2007 | NWFSC |
| 899 | oyster | WA, 2007 | NWFSC |
| 905 | oyster | WA, 2007 | NWFSC |
| 910 | oyster | WA, 2007 | NWFSC |
| 920 | oyster | WA, 2007 | NWFSC |
| 927 | oyster | WA, 2007 | NWFSC |
| 928 | oyster | WA, 2007 | NWFSC |
| 929 | oyster | WA, 2007 | NWFSC |
| 930 | oyster | WA, 2007 | NWFSC |
| 937 | oyster | WA, 2007 | NWFSC |
| 938 | oyster | WA, 2007 | NWFSC |
| 941 | oyster | WA, 2007 | NWFSC |
| 945 | oyster | WA, 2007 | NWFSC |
| 949 | oyster | WA, 2007 | NWFSC |
| 950 | oyster | WA, 2007 | NWFSC |
| 3256 | clinical | WA, 2007 | WDOH-PHL |
| 3259 | clinical | WA, 2007 | WDOH-PHL |
| 3270 | clinical | WA, 2007 | WDOH-PHL |
| 3271 | clinical | WA, 2007 | WDOH-PHL |
| 3316 | clinical | WA, 2007 | WDOH-PHL |
| 3324 | clinical | WA, 2007 | WDOH-PHL |
| 3326 | clinical | WA, 2007 | WDOH-PHL |
| 3328 | clinical | WA, 2007 | WDOH-PHL |
| 3335 | clinical | WA, 2007 | WDOH-PHL |
| 3343 | clinical | WA, 2007 | WDOH-PHL |
| 3355 | clinical | WA, 2007 | WDOH-PHL |
| 3359 | clinical | WA, 2007 | WDOH-PHL |
| 3627 | clinical | WA, 2007 | WDOH-PHL |
| 3631 | clinical | WA, 2007 | WDOH-PHL |
| 3634 | clinical | WA, 2007 | WDOH-PHL |
| 3636 | clinical | WA, 2007 | WDOH-PHL |
| 3644 | clinical | WA, 2007 | WDOH-PHL |
| 3645 | clinical | WA, 2007 | WDOH-PHL |
| 3646 | clinical | WA, 2007 | WDOH-PHL |
| 3651 | clinical | WA, 2007 | WDOH-PHL |
| 3659 | clinical | WA, 2007 | WDOH-PHL |
| 3661 | clinical | WA, 2007 | WDOH-PHL |
| 3689 | clinical | WA, 2007 | WDOH-PHL |
| 48256 | clinical | WA, 1991 | WDOH-PHL |
| 12218 | clinical | WA, 2006 | WDOH-PHL |
| 12225 | clinical | WA, 2006 | WDOH-PHL |
| 12227 | clinical | WA, 2006 | WDOH-PHL |
| 12229 | clinical | WA, 2006 | WDOH-PHL |
| 12234 | clinical | WA, 2006 | WDOH-PHL |
| 12239 | clinical | WA, 2006 | WDOH-PHL |
| 12250 | clinical | WA, 2006 | WDOH-PHL |
| 12251 | clinical | WA, 2006 | WDOH-PHL |
| 12257 | clinical | WA, 2006 | WDOH-PHL |
| 12258 | clinical | WA, 2006 | WDOH-PHL |
| 12259 | clinical | WA, 2006 | WDOH-PHL |
| 12260 | clinical | WA, 2006 | WDOH-PHL |
| 12261 | clinical | WA, 2006 | WDOH-PHL |
| 12280 | clinical | WA, 2006 | WDOH-PHL |
| 12285 | clinical | WA, 2006 | WDOH-PHL |
| 12298 | clinical | WA, 2006 | WDOH-PHL |
| 12307 | clinical | WA, 2006 | WDOH-PHL |
| 12308 | clinical | WA, 2006 | WDOH-PHL |
| 12310 | clinical | WA, 2006 | WDOH-PHL |
| 12315 | clinical | WA, 2006 | WDOH-PHL |
| 12317 | clinical | WA, 2006 | WDOH-PHL |
| 12333 | clinical | WA, 2006 | WDOH-PHL |
| 12353 | clinical | WA, 2006 | WDOH-PHL |
| 12355 | clinical | WA, 2006 | WDOH-PHL |
| 12378 | clinical | WA, 2006 | WDOH-PHL |
| 12383 | clinical | WA, 2006 | WDOH-PHL |
| 12402 | clinical | WA, 2006 | WDOH-PHL |
| 12447 | clinical | WA, 2006 | WDOH-PHL |
| 12601 | clinical | WA, 2006 | WDOH-PHL |
| SPRC10290 | clinical | WA, 1997 | FDA-PRLN |
| 10292 | clinical | WA, 1997 | FDA-PRLN |
| 10296 | clinical | WA, 1997 | FDA-PRLN |
| 10327 | clinical | WA, 1998 | FDA-PRLN |
| 901128 | clinical | ID, 1997 | FDA-PRLN |
| 2006286 | clinical | CT, 1997 | FDA-GCSL |
| 97-029 | environmental | WA, 1997 | FDA-PRLN |
| 97-046a | environmental | WA, 1997 | FDA-PRLN |
| 97-10290 | clinical | WA, 1997 | FDA-PRLN |
| 97-0107 | environmental | WA, 1997 | FDA-PRLN |
| 97-10293 | clinical | WA, 1997 | FDA-PRLN |
| AOC1 | oyster | WA, 1994 | FDA-PRLN |
| AP-14861 | clinical | Bangladesh | FDA-GCSL |
| BE98-2029 | clinical | TX, 1998 | FDA-PRLN |
| NY477 | clinical | NY, 1997 | FDA-GCSL |
| T3937 | clinical | Japan | FDA-GCSL |
| W90A | environmental | WA | FDA-PRLN |
| WR1 | water | WA, 1993 | FDA-PRLN |
| EN2883 | clinical | WA, 2000 | WDOH-PHL |
| EN2910 | clinical | WA, 2000 | WDOH-PHL |
| EN3107 | clinical | WA, 2000 | WDOH-PHL |
| 9401078 | clinical | WA, 1994 | FDA-PRLN |
| EN9701042 | clinical | WA, 1997 | WDOH-PHL |
| EN9701072 | clinical | WA, 1997 | WDOH-PHL |
| EN9701121 | clinical | WA, 1997 | WDOH-PHL |
| EN9701124 | clinical | WA, 1997 | WDOH-PHL |
| EN9701141 | clinical | WA, 1997 | WDOH-PHL |
| EN9701153 | clinical | WA, 1997 | WDOH-PHL |
| EN9701173 | clinical | WA, 1997 | WDOH-PHL |
| EN9701193 | clinical | WA, 1997 | WDOH-PHL |
| EN9901166 | clinical | WA, 1999 | WDOH-PHL |
| EN9901251 | clinical | WA, 1999 | WDOH-PHL |
| EN9901252 | clinical | WA, 1999 | WDOH-PHL |
| EN9901309 | clinical | WA, 1999 | WDOH-PHL |
| EN9901310 | clinical | WA, 1999 | WDOH-PHL |
| HC-01-22 | clinical | WA, 2001 | FDA-GCSL |
| RIMD2210633 | clinical | Japan, 1996 | RIMD-Osaka |
| TX2103 | clinical | TX, 1998 | FDA-GCSL |
| VP80-1B | sediment | WA, 1992 | FDA-PRLN |
| BAC4092 | clinical | NY, 1998 | FDA-GCSL |
| BAC03255 | clinical | NY, 1998 | FDA-GCSL |
| VPHY67 | clinical | Thailand | FDA-GCSL |
| VPHY145 | clinical | Thailand | FDA-GCSL |
| AO24491 | clinical | Bangladesh | FDA-GCSL |
| AP10866 | clinical | Bangladesh | FDA-GCSL |
| AP11243 | clinical | Bangladesh | FDA-GCSL |
| FIHES-98V1-32-4 | clinical | Bangladesh | FDA-GCSL |
| AN2189 | clinical | Bangladesh | FDA-GCSL |
| AN2416 | clinical | Bangladesh | FDA-GCSL |
| HC-06 | environmental | WA | FDA-GCSL |
| 967263412A | clams | Vietnam | FDA-GCSL |
| 96Q | clinical | Gulf Coast, USA | FDA-GCSL |
| ATCC27969 | crustacean | VA, 1969 | ATCC |
| ATCC17802 | clinical | Japan, 1951 | ATCC |
| AQ4037 | clinical | Maldives | FDA-GCSL |

^a^NOAA’s Northwest Fisheries Science Center (NWFSC), Washington Department of Health – Pacific Health Laboratory (WDOH-PHL), Food and Drug Administration’s Pacific Regional Laboratory Northwest (FDA-PRLN), Gulf Coast Seafood Laboratory (FDA-GCSL), Research Institute for Microbial Disease at Osaka University (RIMD-Osaka) and the American Type Culture Collection (ATCC)

Table S1. Description of the *V. parahaemolyticus* isolates (N = 167) included in this investigation including source (clinical and environmental), location and date of isolation, and laboratory housing or providing the specific isolate.
