# Supplementary material for: Population Structure of Clinical and Environmental Vibrio parahaemolyticus from the Pacific Northwest Coast of the United States
Source: PLoS One. 2013 Feb 7;8(2):e55726. doi: 10.1371/journal.pone.0055726 (PMC3567088; doi:10.1371/journal.pone.0055726)
Supplement: Table S2 — REP-PCR results. Summary of REP-PCR results (N = 167 isolates) organized by REP group including the number of isolates in each group and the genotype (tdh, trh, ureR) of each REP-PCR group. (DOC) [file pone.0055726.s002.doc]

Table S2. REP-PCR results.

| ***V. parahaemolyticus* isolates** | **N** | **REP-PCR** | **Genotype** |
| --- | --- | --- | --- |
| SRPC10290, 12298, 12355, 12310, 12383, 3335, 3270, 3271, 3326, 3256, 3343, 3316, 3324, 12229, 97029, 10296, W90A, 12315, 12317, 837, 846, 12307, 12308, 12258, 12280, 12285, 12261, 12257, 12333, 12378, 12260, 12225, 12218, 12250, 12227, 12251, 12234, 12239, 12353, 12259, EN9701224, EN9701193, EN2910, EN9701173, EN9701141, EN9701053, EN9901309, EN9901252, EN9901251, EN9901166, 97-029 | 51 | 1 | *tdh*^+^*, trh*^+^, *ure*R^+^ |
|  |  |  |
|  |  |  |
|  |  |  |
|  |  |  |
| RIMD2210633, 863, 743, VP747, 752, 571, 861, 782, 605, 930, 920, 929, 941, 783, 658, VP551, 668, 661, 671, 688, BE982029, AP14861, TX2103, BAC4092, BAC03255, VPHY67, VPHY145, AO24491, AP11243, FIHES-98V1-32-4, AN2189, AN2416, 928, 927, 910, 899, 905, 950, 604, 588, 606, 587, 586, 584, 585, 864, 865 | 47 | 2 | *tdh*^+^*, trh*^±^, *ure*R^±^ |
|  |  |  |
|  |  |  |
|  |  |  |
| 949, 945, 937, 938 | 4 | 3 | *tdh*^+^, *trh*^-^, *ure*R^-^ |
| 27, 50, 260 | 3 | 4 | *tdh*^+^, *trh*^+^, *ure*R^+^ |
| 31, 49, 55 | 3 | 5 | *tdh*^-^, *trh*^-^, *ure*R^-^ |
| 43, VP766, 7 | 3 | 6 | *tdh*^-^, *trh*^-^, *ure*R^-^ |
| 361, 38 | 2 | 7 | *tdh*^-^, *trh*^-^, *ure*R^-^ |
| 97-046a, 97-10290, 12601, HC-01-22, EN3107, 3645, 3644, 3636, 3634, 3651, EN9701072, EN9701042 | 12 | 8 | *tdh*^+^, *trh*^+^, *ure*R^+^ |
|  |
| 3328, 3355, 3259 | 3 | 9 | *tdh*^-^, *trh*^+^, *ure*R^+^ |
| 901128, AOC1 | 2 | 10 | *tdh*^-^, *trh*^-^, *ure*R^-^ |
| 3627, 3659, 3631, 3689, 3646 | 5 | 11 | *tdh*^-^, *trh*^+^, *ure*R^+^ |
| EN9901310, 9401078 | 2 | 12 | *tdh*^+^, *trh*^+^, *ure*R^+^ |
| EN9701121, 10292 | 2 | 13 | *tdh*^+^, *trh*^+^, *ure*R^+^ |
| VP80-1B, 48256 | 2 | 14 | *tdh*^-^*, trh*^+^, *ure*R^+^ |
| 67 | 1 | 15 | *tdh*^-^*,trh*^-^*, ure*R^-^ |
| WR1 | 1 | 16 | *tdh*^-^, *trh*^-^, *ure*R^-^ |
| 204 | 1 | 17 | *tdh*^-^, *trh*^+^, *ure*R^+^ |
| NY477 | 1 | 18 | *tdh*^+^, *trh*^-^, *ure*R^-^ |
| 97-0107 | 1 | 19 | *tdh*^-^, *trh*^-^, *ure*R^-^ |
| 2006286 | 1 | 20 | *tdh*^+^, *trh*^+^, *ure*R^+^ |
| T3937 | 1 | 21 | *tdh*^+^, *trh*^+^, *ure*R^+^ |
| 2 | 1 | 22 | *tdh*^-^, *trh*^-^, *ure*R^-^ |
| 32 | 1 | 23 | *tdh*^-^, *trh*^-^, *ure*R^-^ |
| 3359 | 1 | 24 | *tdh*^-^, *trh*^-^, *ure*R^-^ |
| 805 | 1 | 25 | *tdh*^-^, *trh*^-^, *ure*R^-^ |
| 12402 | 1 | 26 | *tdh*^+^*, trh*^+^*,* *ure*R^+^ |
| EN2883 | 1 | 27 | *tdh*^+^*, trh+,* *ure*R^+^ |
| HC-06 | 1 | 28 | *tdh*^-^*, trh*^+^*,* *ure*R^+^ |
| 3661 | 1 | 29 | *tdh*^-^*, trh*^-^*,* *ure*R^-^ |
| 967263412A | 1 | 30 | *tdh*^-^*, trh*^-^*,* *ure*R^-^ |
| 96Q | 1 | 31 | *tdh*^-^*, trh*^+^*,* *ure*R^+^ |
| AP-10866 | 1 | 32 | *tdh*^+^*, trh*^+^*,* *ure*R^+^ |
| 10327 | 1 | 33 | *tdh*^+^*, trh*^+^*,* *ure*R^+^ |
| ATCC27969 | 1 | 34 | *tdh*^+^, *trh*^-^, *ure*R^-^ |
| ATCC17802 | 1 | 35 | *tdh*^+^, *trh*^-^, *ure*R^-^ |
| AQ4037 | 1 | 36 | *tdh*^+^, *trh*^-^, *ure*R^-^ |
| 12447 | 1 | 37 | *tdh*^+^, *trh*^+^, *ure*R^+^ |
| 197 | 1 | 38 | *tdh*^-^, *trh*^-^, *ure*R^-^ |
| 6 | 1 | 39 | *tdh*^+^, *trh*^-^, *ure*R^-^ |

Table S2. Summary of REP-PCR results (N = 167 isolates) organized by REP group including the number of isolates in each group and the genotype (*tdh*, *trh*, ureR) of each REP-PCR group.
