# Supplementary material for: Population Structure of Clinical and Environmental Vibrio parahaemolyticus from the Pacific Northwest Coast of the United States
Source: PLoS One. 2013 Feb 7;8(2):e55726. doi: 10.1371/journal.pone.0055726 (PMC3567088; doi:10.1371/journal.pone.0055726)
Supplement: Table S3 — MLST and eBURST results. MLST results (N = 77 isolates) organized by sequence type (ST) including the determination of whether a given ST is part of a clonal complex (CC), group (G) or singleton (S) as determined by eBURST. (DOC) [file pone.0055726.s003.doc]

Table S3. MLST and eBURST results.

| ***V. parahaemolyticus* isolates** | **N** | **Sequence type** | **eBURST** | **Genotype** |
| --- | --- | --- | --- | --- |
| VP551, 571, 605, 658, 668, 743, VP747, 752, 782, 783, 861, 863, 920, 929, 930, 941, AP-14861, BE98-2029, RIMD2210633, TX2103 | 20 | 3 | CC3 | *tdh*^+^*, trh*^±^, *ure*R^±^ |
|  |  |  |  |
| SPRC10290, 10296, 12298, 12310, 12315, 12317, 12355, 12383, 12478, 3256, 3270, 3271, 3324, 3326, 837, 846, 97-029, EN2910, EN9701173, EN9901310 | 20 | 36 | G36 | *tdh*^+^*, trh*^+^, *ure*R^+^ |
|  |  |  |  |
|  |  |  |  |
| 12601, 3644, 97-046a, 97-10290, EN9701072, HC-01-22 | 6 | 43 | S | *tdh*^+^, *trh*^+^, *ure*R^+^ |
| 260, 27, 50, 765 | 4 | 34 | CC34 | *tdh*^+^, *trh*^+^, *ure*R^+^ |
| 3259, 3328, 3355 | 3 | 65 | S | *tdh*^-^, *trh*^+^, *ure*R^+^ |
| 31, 49, 55 | 3 | 137 | S | *tdh*^-^, *trh*^-^, *ure*R^-^ |
| 901128, AOC1 | 2 | 135 | G | *tdh*^-^, *trh*^-^, *ure*R^-^ |
| 361, 38 | 2 | 138 | S | *tdh*^-^, *trh*^-^, *ure*R^-^ |
| 3631, 3646 | 2 | 417 | S | *tdh*^-^, *trh*^+^, *ure*R^+^ |
| EN9701121 | 1 | 50 | CC50 | *tdh*^+^, *trh*^+^, *ure*R^+^ |
| W90A | 1 | 59 | G | *tdh*^+^, *trh*^+^, *ure*R^+^ |
| NY477 | 1 | 88 | CC* | *tdh*^+^, *trh*^-^, *ure*R^-^ |
| 97-0107 | 1 | 131 | S | *tdh*^-^, *trh*^-^, *ure*R^-^ |
| VP766 | 1 | 133 | CC322 | *tdh*^-^, *trh*^-^, *ure*R^-^ |
| WR1 | 1 | 134 | S | *tdh*^-^, *trh*^-^, *ure*R^-^ |
| 6 | 1 | 136 | S | *tdh*^+^, *trh*^-^, *ure*R^-^ |
| 197 | 1 | 139 | G | *tdh*^-^, *trh*^-^, *ure*R^-^ |
| VP80-1B | 1 | 141 | S | *tdh*^-^, *trh*^+^, *ure*R^+^ |
| 32 | 1 | 142 | S | *tdh*^-^, *trh*^-^, *ure*R^-^ |
| T3937 | 1 | 143 | S | *tdh*^+^, *trh*^+^, *ure*R^+^ |
| 43 | 1 | 322 | CC322 | *tdh*^-^, *trh*^-^, *ure*R^-^ |
| 805 | 1 | 323 | S | *tdh*^-^, *trh*^-^, *ure*R^-^ |
| 2006286 | 1 | 418 | CC110 | *tdh*^+^, *trh*^+^, *ure*R^+^ |
| 204 | 1 | 416 | S | *tdh*^-^, *trh*^+^, *ure*R^+^ |

^a^Clonal complex (CC), Group (G) and Singleton (S)

^b^CC named after the predicted founding sequence type and based on analysis of all allelic profiles in the MLST database

^c^CC* indicates a clonal complex with multiple candidate founders

Table S3. MLST results (N = 77 isolates) organized by sequence type (ST) including the determination of whether a given ST is part of a clonal complex (CC), group (G) or singleton (S) as determined by eBURST.
